# Periostin attenuates tumor growth by inducing apoptosis in colitis-related colorectal cancer

**DOI:** 10.18632/oncotarget.25026

**Published:** 2018-04-13

**Authors:** Yusuke Shimoyama, Keiichi Tamai, Rie Shibuya, Mao Nakamura, Mai Mochizuki, Kazunori Yamaguchi, Yoichi Kakuta, Yoshitaka Kinouchi, Ikuro Sato, Akira Kudo, Tooru Shimosegawa, Kennichi Satoh

**Affiliations:** ^1^ Division of Cancer Stem Cell, Miyagi Cancer Center Research Institute, Natori, Japan; ^2^ Division of Molecular and Cellular Oncology, Miyagi Cancer Center Research Institute, Natori, Japan; ^3^ Department of Gastroenterology, Tohoku University Graduate School of Medicine, Sendai, Japan; ^4^ Department of Pathology, Miyagi Cancer Center, Natori, Japan; ^5^ Department of Biological Information, Tokyo Institute of Technology, Yokohama, Japan

**Keywords:** periostin, colorectal cancer, colitis, apoptosis

## Abstract

Inflammatory bowel diseases, which are multifactorial autoimmune colitis diseases, are occurring with increasing prevalence. One of the most serious complications of these diseases is colorectal cancer. Here we investigated the role of periostin (Postn), a matricellular protein that interacts with various integrin molecules on the cell surface, in colitis-induced colorectal cancer. Immunohistochemistry of mouse and human colorectal cancer samples revealed that Postn was expressed in the stroma and was upregulated in close proximity to the cancer cells. The colonic tumorigenesis in an inflammation-related colon carcinogenesis mouse model was increased in Postn knock-out (Postn^−/−^) mice compared to Postn^+/+^ mice. Although no difference was found in the degree of colitis between Postn^+/+^ and Postn^−/−^ mice, Postn inhibited tumor growth and induced the apoptosis of mouse rectal cancer cells *in vitro*. Furthermore, fewer apoptotic colorectal cancer cells were observed in Postn^−/−^ than in Postn^+/+^ mice. These data suggested that Postn has an anti-tumor effect on colitis-induced colorectal cancer.

## INTRODUCTION

Ulcerative colitis (UC) is a common type of inflammatory bowel disease (IBD). IBD is associated with chronic inflammation of the digestive tract, resulting in abdominal pain, persistent diarrhea, and hematochezia. The prevalence of UC in Japan is 63.6 individuals per 100,000 in 2005 [1]. Anti-tumor necrosis factor (TNF) agents are effective for attaining and maintaining IBD remission. In developed countries, colorectal cancer (CRC) is one of the leading non-smoking-related cancers. This malignancy is one of the most serious complications of IBD, including UC [2], and the risk of CRC increases with the severity and duration of the IBD. Surgical resection is the primary treatment for CRC. Although research has focused on finding novel agents targeting the CRC tumor's angiogenic activity and cell growth (i.e., VEGF and EGF), these patients often die from recurrence and dissemination of the cancer soon after surgery. Thus, new strategies for improving the prognosis and individualized treatment of this cancer are urgently needed.

Periostin (Postn) is a secreted matrix *N*-glycoprotein with four internal repeat regions that containing integrin- and glycosaminoglycan-binding sequences [3]. The binding of Postn to integrins initiates cross-talk between the integrins and receptor tyrosine kinases like EGF at the plasma membrane. These interacting molecules co-activate the serine threonine Akt and Erk cell signaling pathway, which modulates cell motility, proliferation, and survival.

Postn is expressed in cancer-associated fibroblasts of colon, breast, lung, pancreatic, and stomach cancer [4, 5], and plays critical roles in the epithelial-mesenchymal transition of tumor cells [6, 7]. Postn is also associated with tumor invasiveness and a poor prognosis in several malignancies [8–10]. A high expression of Postn in tumor stroma was found to be an independent prognostic indicator of poor 5-year cancer-specific survival and poor 5-year progression-free survival. Postn is also up-regulated in lung inflammatory diseases such as asthma and idiopathic pulmonary fibrosis and has an overarching role in lung tissue remodeling and repair [11]. Thus, Postn interacts with multiple signaling cascades to modulate the expression of several genes, and has multiple functions in inflammatory diseases and tumors [3]. However, little is known about the role of Postn in colitis-induced CRC.

We demonstrate here that Postn inhibits tumor growth by inducing apoptosis in colorectal adenocarcinoma. Using Postn knock-out mice, we revealed that Postn plays critical roles in the development of dextran sulfate sodium (DSS)-induced CRC, but not in DSS-induced colitis.

## RESULTS

### Periostin expression surrounding tumor cells

**Figure 1:**
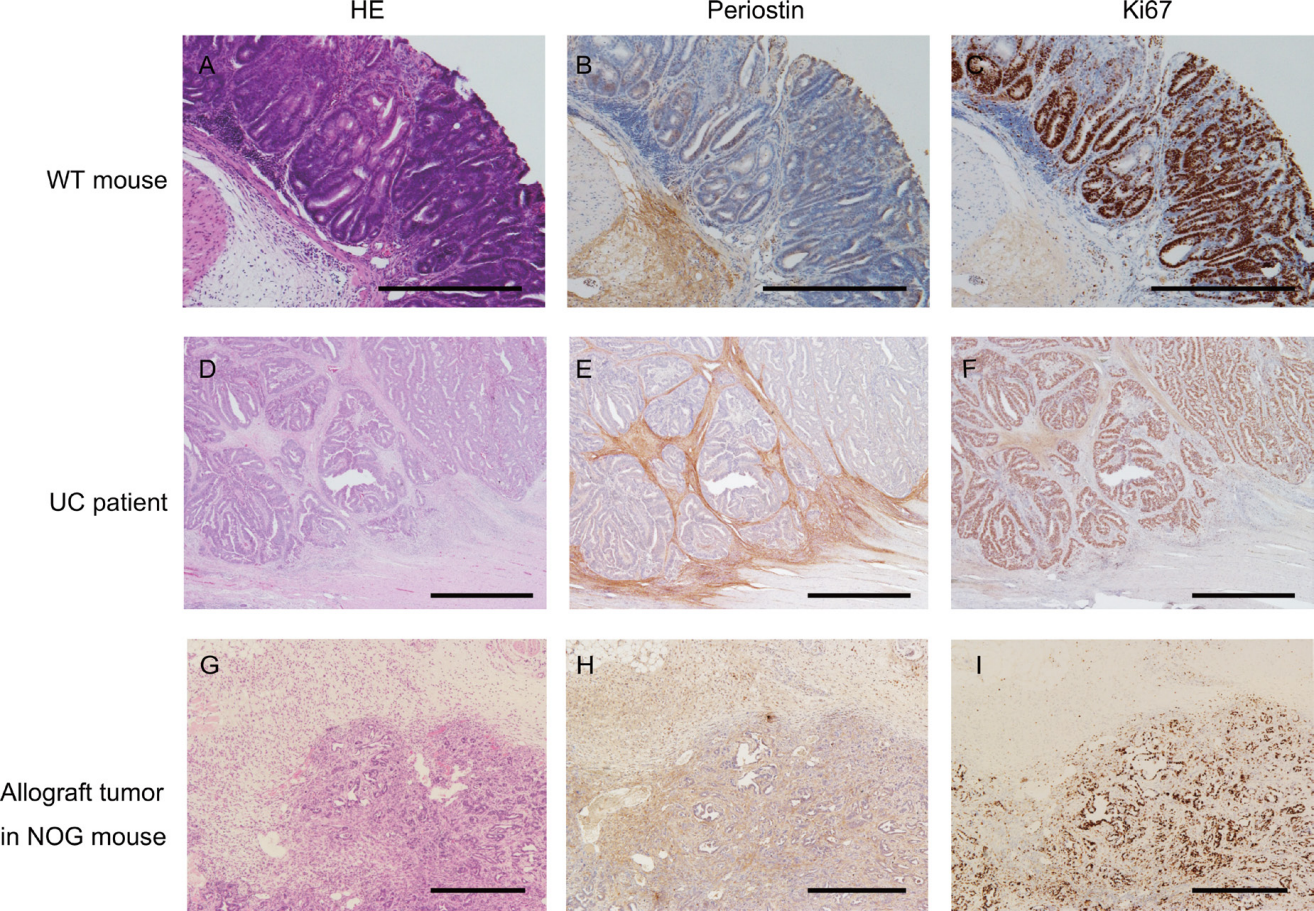
Periostin expression in colorectal cancer Colitis-induced colorectal cancer in mice (**A** to **C**), colorectal cancer of ulcerative colitis patients (**D** to **F**), and an allograft of CMT93 cells in NOG mice were stained with hematoxylin and eosin (A, D, and **G**), and with anti-Postn (**B**, **E**, and **H**), and anti-Ki67 (C, F, and **I**) antibodies. WT, wild-type; UC, ulcerative colitis.

To examine the localization of Postn in colorectal cancer, we first performed IHC for Postn in the AOM/DSS-induced colitis-associated colorectal cancer mouse model. Postn was slightly expressed in epithelial cells, but was mostly localized to the stroma of the submucosal area, especially in the invasive front (Figure 1A and 1B). In addition, IHC showed that Ki67 was expressed adjacent to the submucosal area (Figure 1C). In human ulcerative colitis-related colorectal cancer samples, Postn was expressed around the tumor cells, and Ki67 was expressed in almost all of the tumor cells (Figure 1D to 1F). We also implanted CMT93 cells into NOD/Shi-scid-IL2R γ null (NOG) mice, and four weeks later, resected the tumor and stained it with anti-Postn and anti-Ki67 antibodies. Similar to the mouse model and human tissues, Postn was expressed in the submucosal tissue near the CMT93 cells (Figure 1G to 1I). These data indicated that Postn is expressed adjacent to and upregulated by CRC tumor cells.

### Periostin inhibits the CRC formation resulting from AOM/DSS-induced colitis

**Figure 2:**
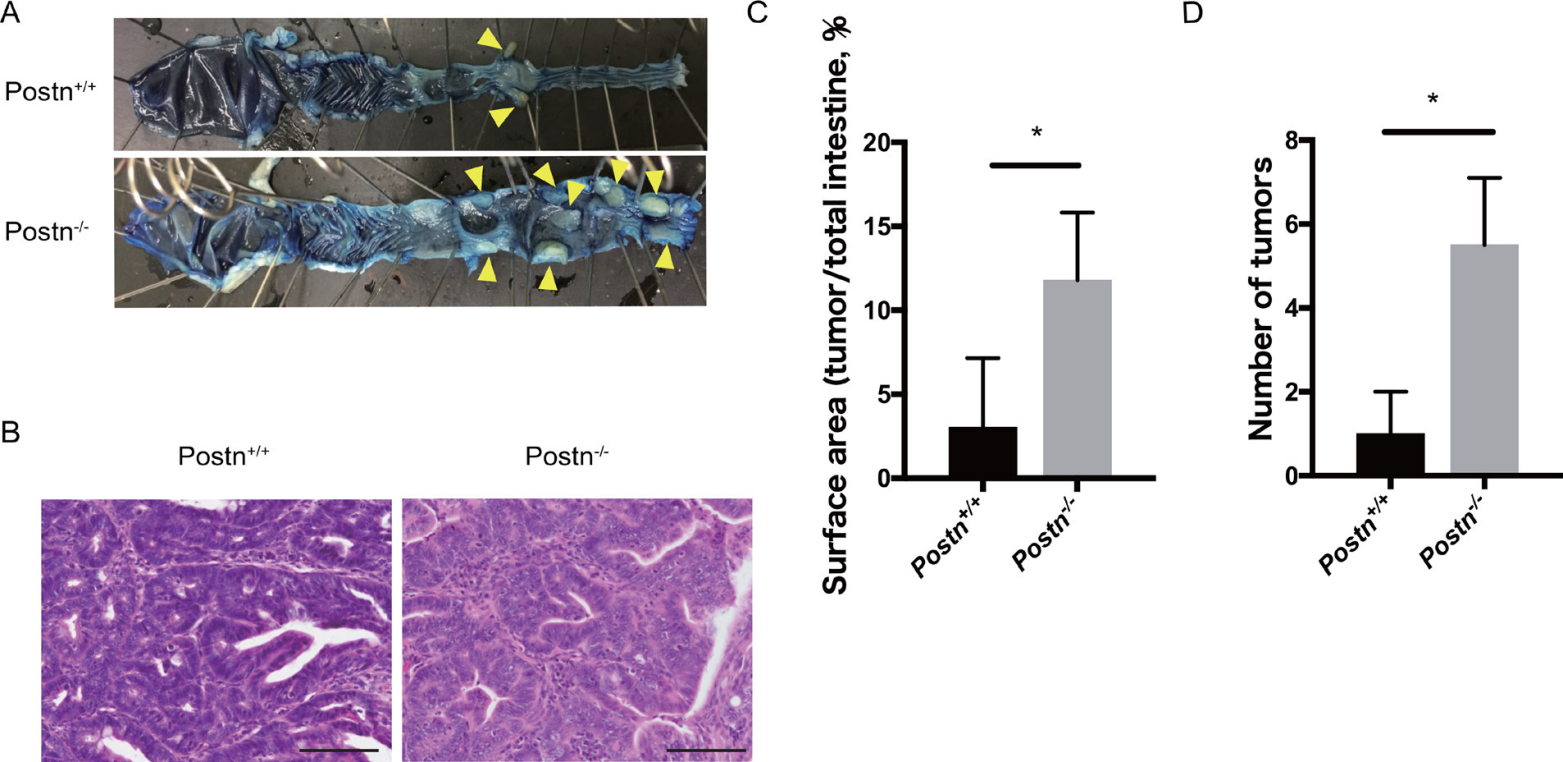
Colitis-induced colorectal cancer development was facilitated in Postn−/− mice (**A**) Macroscopic images of the large intestine from Postn^+/+^ and Postn^−/−^ AOM/DSS-model mice. Arrowheads indicate colorectal tumors. (**B**) Hematoxylin and eosin staining of the adenocarcinoma in Postn^+/+^ and Postn^−/−^ mice. Bar, 100 μm. (**C**) Quantification of the tumor surface area. The surface area was measured using ImageJ software. ^*^P < 0.05. (**D**) Number of colorectal tumors in Postn^+/+^ and Postn^−/−^ mice. Postn, Periostin. ^*^P < 0.01.

To clarify the role of Postn in colitis-related CRC, we analyzed the AOM/DSS-induced tumorigenesis using Postn knock-out mice. At 140 days after AOM/DSS treatment, mice were sacrificed and analyzed for CRC. Colorectal adenocarcinoma was generated in both Postn^+/+^ and Postn^–/–^ mice (Figure 2A and 2B). Notably, however, both the tumor surface area and the number of tumors were increased in the Postn^–/–^ mice (Figure 2C and 2D). These data indicated that Postn has an inhibitory effect against colitis-induced colorectal cancer.

### Periostin does not affect inflammation

**Figure 3:**
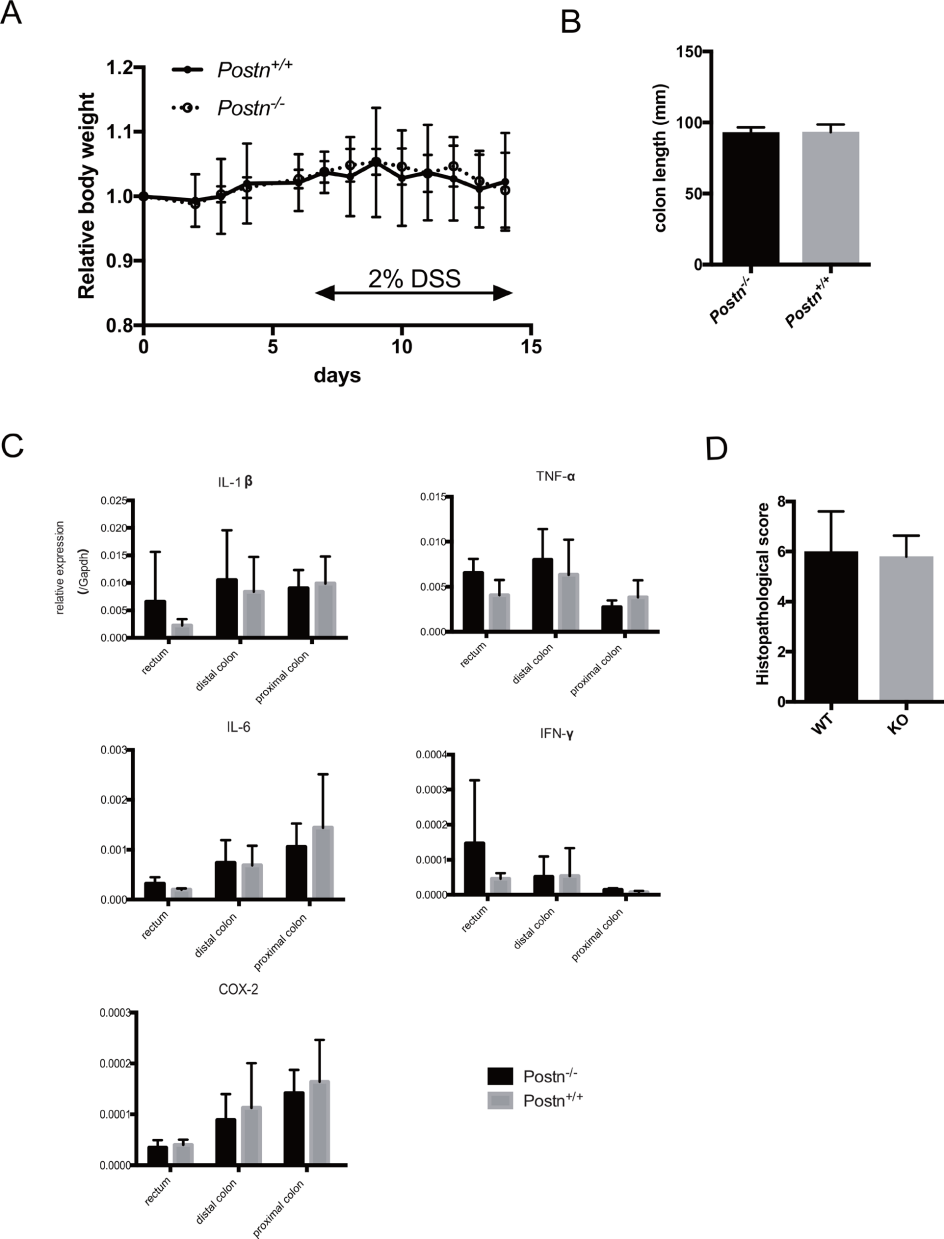
Periostin does not mediate intestinal inflammation in AOM/DSS models (**A**) Body weight change after AOM administration. (**B**) Length of extracted large intestine 14 days after AOM administration. (**C**) Expression of inflammation-related genes in the large intestine measured by real-time PCR. (**D**) Histopathological score of the large intestine. Postn, Periostin.

We next evaluated whether Postn affects the inflammation process in the AOM/DSS-induced colitis model. We found no differences between the Postn^+/+^ and Postn^–/–^ mice in the daily body weight after AOM/DSS treatment (Figure 3A) and in the colon length 14 days after AOM/DSS treatment (Figure 3B). We also measured the expression level of several cytokines and COX-2, which are mediators induced by inflammation. No significant differences were observed in any region of the large intestine between the Postn^+/+^ and Postn^–/–^ mice (Figure 3C). We also found no difference in the histopathological score of the large intestine between the Postn^+/+^ and Postn^–/–^ mice. These data indicated that Postn does not affect the inflammation process induced by AOM/DSS.

### Postn induces apoptosis and inhibits cell growth

**Figure 4:**
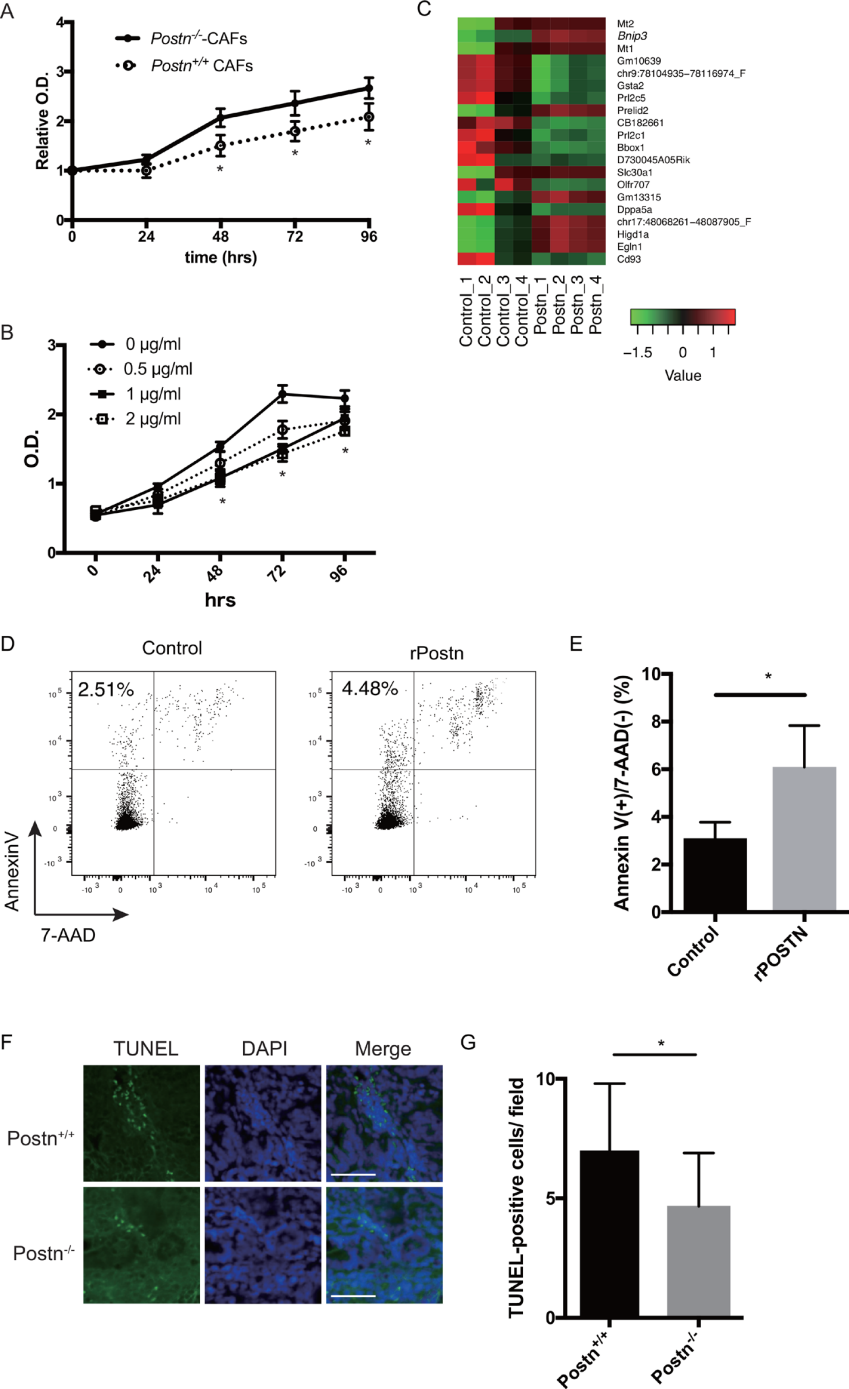
Periostin induces apoptosis in colorectal cancer (**A**) Proliferation of CMT93 cells cultured with the conditioned medium collected from Postn^+/+^ or Postn^−/−^ cancer-associated fibroblasts (CAFs) (cultured for 48 h), assessed by MTT assay. ^*^P < 0.05. (**B**) Proliferation of CMT93 cells under rPostn stimulation assessed by MTT assay. ^*^P < 0.05. (**C**) Heatmap showing the expression profile of 20 genes in Postn-treated (Postn_1–4) and control (Control_1–4) cells, determined by microarray analysis using the weighted average difference (WAD) algorithm. Red indicates higher and green indicates lower abundance (Z-score). (**D**) Representative dot plots of AnnexinV and 7-AAD staining in Postn-treated or control CMT93 cells. (**E**) Frequency of early apoptotic cells (AnnexinV(+) and 7-AAD(–)). ^*^P < 0.05. (**F**) Representative TUNEL assay images in Postn^+/+^ and Postn^−/−^ mice. Bar, 50 μm. (**G**) Quantification of TUNEL-positive cells. *n* = 15. Postn, Periostin. ^*^P < 0.05.

We speculated that Postn directly inhibits the proliferation of tumor cells in the colitis-induced colon adenocarcinoma. To test this possibility, we examined the effect of Postn on tumor cells *in vitro*. Because Postn was secreted mainly from fibroblasts (Figure 1), we established CRC-associated fibroblast cell lines (CAF) from the large intestine of Postn^–/–^ and Postn^+/+^ mice (Supplementary Figure 1). To investigate the effect of these CAFs on cancer cells, we cultured CMT93 cells with the conditioned medium of the Postn^+/+^ and Postn^–/–^ CAFs. By day 2, the number of CMT93 cells cultured with the conditioned medium of Postn^+/+^ CAFs was decreased compared with the cells cultured with Postn^–/–^ medium (Figure 4A). To clarify the role of Postn on cancer cell proliferation, we examined the cell proliferation under rPostn stimulation. By day 2, the proliferation rate was lower under rPostn stimulation (Figure 4B). We also examined the cell proliferation using the colon-26 cell line, which is derived from mouse rectal cancer, and obtained similar results (Supplementary Figure 2A). To test whether the intracellular growth signaling was altered in the presence of Postn, we examined the phosphorylation levels of Erk and Akt. However, no significant difference was found in these phosphorylations (Supplementary Figure 3). We then performed a microarray analysis to identify the differentially expressed genes under rPostn stimulation. Based on a literature search of the 20 genes that were most differentially expressed as identified by the WAD algorithm (Figure 4C), we focused on Bnip3 (Bcl2-interaciting protein 3), because it was previously identified as a key factor in apoptosis. To examine the apoptosis induction by Postn, we investigated the expression of annexinV under rPostn stimulation by flow cytometry. The proportion of annexinV-positive and 7-AAD-negative cells, which indicates early apoptosis, was increased by rPostn stimulation (Figure 4D and 4E). We also examined the cell proliferation using the colon-26 cell line, and obtained similar results (Supplementary Figure 2B). We then analyzed the induction of apoptosis in the mouse colitis-induced colon adenocarcinoma by TUNEL (TdT-mediated dUTP nick end labeling) assay, and found that the TUNEL-positive cells were increased in Postn^+/+^ compared with Postn^–/–^ mouse tumors (Figure 4F and 4G). These data indicated that Postn plays critical roles in apoptosis signaling and inhibits tumor growth.

## DISCUSSION

Our IHC analysis of human CRC showed that Postn was localized to the stroma near the invasive front. In the mouse CRC allograft model, the Postn expression was increased surrounding the cancer cells. A previous report demonstrated that the serum levels of Postn in CRC patients are significantly elevated compared to those in healthy and benign colorectal polyps and adenomas, and that the preoperative serum Postn levels are significantly higher than those in the same patients after tumor removal [16]. TGF-ß promotes the secretion of Postn [17], and TGF-ß secreted by epithelial cancer cells exerts a paracrine influence on stromal cells, resulting in an increased production of extracellular matrix [18]. Collectively, we speculate that cancer cells, but not normal epithelium of the intestine, secrete factors such as TGF-ß, which induce Postn secretion in cancer-associated fibroblasts, and that Postn mediates an anti-tumor effect in colitis-induced CRC. Further study will be required to elucidate the molecular mechanisms of the interaction between cancer cells and CAFs.

We demonstrated that Postn induces the apoptosis of cancer cells *in vitro* and *in vivo*. Microarray analysis revealed that Bnip3, which induces a cell death characterized by the localization of Bnip3 to the mitochondria, is increased by rPostn stimulation. *BNIP3* is a HIF1A target gene that is induced by hypoxia but is also transcriptionally regulated by RB1-E2F1, TP53, FOXO3, NF-κB, and other tumor relevant transcription factors [19]. To clarify the role of Bnip3 in apoptosis, we examined the proliferation of CMT93 cells using siRNA against *Bnip3*, but no significant difference was found between the Bnip3-knock down and control cells (data not shown), suggesting that redundant pathways exist for the Postn-dependent apoptosis.

In this study, we also demonstrated that the degree of inflammation induced by AOM/DSS treatment was not significantly different between Postn^+/+^ and Postn^–/–^ mice. In contrast, a recent study suggested that the oral administration of 4% DSS induced severe colitis with weight loss and a shortened colon in wild-type mice, but not in Postn^–/–^ mice [20]. Since the mice in our study received 2% DSS in the drinking water, and almost no weight loss was observed even in the Postn^+/+^ mice in our study, the degree of inflammation was relatively weak, and Postn appeared to have little effect on the colitis under these conditions.

We demonstrated that Postn induces apoptosis and inhibits tumor growth. Previous reports suggested that Postn also promotes apoptotic cell death in Dupuytren's disease, which is compatible with our present findings [21]. In contrast, in another study, Postn derived from colonic fibroblasts or recombinant Postn (up to 100 ng/ml) was demonstrated to promote the proliferation of human CRC cells [10]. In addition, Postn overexpression in the tumor stroma was shown to be a poor prognostic indicator of CRC [10, 22]. In addition, it was previously reported that a low concentration (150 ng/ml) of Postn reduces the migration of pancreatic cancer cells, while a high concentration (1,000 ng/ml) promotes it, suggesting that Postn has a biphasic effect. Since we used 500–2,000 ng/ml Postn, which was a relatively high concentration compared to the previous report [10], Postn may also have biphasic effects on CRC cell growth.

In conclusion, we demonstrated that Postn is upregulated near mouse and human CRC cells, and that Postn knock-out mice exhibited increased colitis-induced colon cancer development. We further showed that Postn promoted cancer cell apoptosis *in vivo* and *in vitro*. These findings suggested that Postn plays critical roles in the regulation of colitis-induced CRC.

## MATERIALS AND METHODS

### Ethics statements

This study was conducted according to the principles expressed in the Declaration of Helsinki and was approved by the Ethics Committees of the Miyagi Cancer Center Research Institute (Natori, Japan) and Tohoku University Graduate School of Medicine (Sendai, Japan). The animal experimental protocols were approved by the Miyagi Cancer Center Animal Care and Use Committee (MCC-AE-2016-7).

### Cell lines

The CMT93 cell line (mouse rectal cancer) was purchased from American Type Culture Collection. CMT93 cells were maintained in DMEM supplemented with 10% fetal bovine serum (FBS) and 1% penicillin-streptomycin.

### Animals

C57BL/6 Postn knock-out (Postn^–/–^) mice were generated using Cre recombination to create the null allele as described previously [12].

### Inflammation-related mouse colon carcinogenesis model

The inflammation-related mouse colon carcinogenesis model was generated as described previously [13]. In brief, Postn^+/+^ and Postn^–/–^ mice (8–10 weeks old) were given a single intraperitoneal injection of azoxymethane (AOM) (10 mg/kg body weight, Sigma-Aldrich). Starting 7 days after the injection, the animals received 2% DSS (36–50 kDa, MP Biomedicals) in the drinking water for 7 days, and then no further treatment for 126 days. On day 140 after the AOM injection, the mice were sacrificed and the large intestine was resected, fixed with 10% buffered formalin, cut open longitudinally along the main axis, and washed with saline. Macroscopic images were obtained after indigo carmine spraying (1 mg/ml). The surface area of the total colon and tumor (over 2 mm in diameter) was measured using ImageJ. For the evaluation of AOM/DSS-induced colitis, the mice were sacrificed 14 days after the AOM injection. The length of the resected intestine was measured (from the ileocecal junction to the anal verge) and cut into three sections: the rectum, distal/ proximal large intestine.

### Establishment of cancer-associated fibroblasts

Tumors were removed from CRC-induced mice, then the normal colon tissue near the tumor was immediately resected, washed with PBS three times, cut into pieces, and seeded into a 10-cm dish with RPMI medium supplemented with 10% FBS, 0.1% penicillin-streptomycin-amphotericin (Wako), and 0.05% gentamicin. When fibroblast outgrowth was observed, the culture medium was replaced with RPMI supplemented with 10% FBS and 1% penicillin-streptomycin.

### Colorectal cancer allograft model

CMT93 cells were implanted subcutaneously into NOD/Shi-scid-IL2R γ null (NOG) mice. After 28 days, the mice were sacrificed and the subcutaneous tumor was resected and fixed in 10% buffered formalin.

### Tissue specimens

Tissue specimens were obtained from 13 patients with ulcerative colitis-related colorectal cancer from 1977 to 2016 at Tohoku University Hospital. All of the patients provided written informed consent.

### Real time PCR

Mouse large intestine was divided into three parts (rectum, distal colon, and proximal colon) based on the macroscopic pattern of the mucosal folds. The total RNA was isolated from each part of the intestine using an RNeasy Mini Kit (Qiagen) according to the manufacturer's protocol, then 500 ng of the total RNA was reverse transcribed using a PrimeScript 1st strand cDNA Synthesis Kit (Takara). Real-time PCR was performed using the Brilliant III Ultra-Fast SYBR Green QPCR Master Mix (Agilent Technologies). The primer sequences used in this study were shown as follows: TNF-α, F- CGTCAGCCGATTTGCTATCT, R- CGGACTCCGCAAAGTCTAAG; IFN-γ, F- GCTCTGAG ACAATGAACGCT, R- AAAGAGATAATCTGCTCTGC; COX-2, F- TGCCTGGTCTGATGATGTATGCCA, R- AGTAGTCGCACACTCTGTTGTGCT, IL-1β, F- ACTC ATTGTGGCTGTGGAGA, R- TTGTTCATCTCGGAGC CTGT; IL-6, F- GTTGCCTTCTTGGGACTGATG, R- GT ATAGACAGGTCTGTTGGGAG; GAPDH, F- AGGTAGG TGTGAACGGATTTG, R- TGTAGACCATGTAGTTGA GGTCA. Relative gene expression values were calculated using the ΔΔCT method.

### Flow cytometry analysis

CMT93 cells were cultured with DMEM containing 0.1% FBS and 2 μg/ml recombinant Postn for 72 hours. Cells were harvested with 0.25% trypsin and stained with FITC-conjugated anti-annexin V (Biolegend) and 7-AAD (Sigma Aldrich). The cells were analyzed with a FACSCanto II.

### Terminal deoxynucleotidyltransferase-mediated dUTP biotin nick-end labeling (TUNEL) assays

Paraffin-embedded 5-μm sections of tissue samples were deparaffinized, and terminal transferase labeling of the fragmented DNA was performed with an *in situ* cell death detection kit (Fluorescein; Roche, Indianapolis, IN), according to the assay protocol of the kit. Five randomly selected areas were observed by confocal laser microscopy (Nikon A1), and both the DAPI- and TUNEL-positive cells were counted.

### Immunohistochemistry

Immunohistochemistry (IHC) for Postn was performed using the Ventana Discovery automation system (Roche, Switzerland) according to the manufacturer's protocol. A rabbit polyclonal anti-Periostin antibody (ab14041, 1: 2,000, Abcam, UK) was used according to the manufacturer's instructions on 3-μm sections. Anti-Ki67 (clone 30-9, Roche) staining was also performed using the same system.

### Western blotting

Cells (1 × 10^5^) were washed once with PBS (without calcium), lysed in 100 μl of SDS-loading buffer (100 mM Tris-Cl pH 6.8, 4% sodium dodecyl sulfate, 0.2% bromophenol blue, 20% glycerol, 2% ß-mercaptoethanol) and sonicated for 5 min. The samples were boiled for 5 min and then subjected to SDS-PAGE. The separated proteins were transferred onto a PVDF membrane (Millipore, Billerica, MA), which was then incubated with 1: 1,000-diluted primary antibody and then with HRP-conjugated anti-mouse or anti-rabbit antibody (Cell Signaling Technology) as recommended by the manufacturers. Primary antibody binding was detected using a Clarity Western ECL substrate (Bio Rad, Hercules, CA), and images were captured with a CCD camera (Fuji Film, Tokyo, Japan). The following primary antibodies were used: anti-phospho-p44/42 MAPK (ERK1/2, Thr202/Tyr204) (20G11, Cell Signaling Technologies, Danvers, MA, USA), anti-ERK (137E5, Cell Signaling), anti-phospho-Akt (T308) and anti-Akt pan (C31E5E and C67E7, Cell Signaling), anti-phospho-NF-κB p65 (S536) and anti-NF-κB p65 (93H1 and D14E12, Cell Signaling), anti-IκBα (L35A5, Cell Signaling), anti-pIκBα (Ser32, 14D4, Cell Signaling) and anti-α-tubulin (B-5-1-2, Santa Cruz Biotechnology, Dallas, TX, USA).

### MTT assay

A total of 5000 CMT93 cells were plated in 0.1 ml DMEM medium supplemented with 0.1% FBS and recombinant periostin (rPostn, R&D systems, MN) (0–2 μg/ml) in a 96-well plate. At the indicated times, a Cell Counting Kit (Dojindo) was used according to the manufacturer's protocol. The absorbance at 450 nm was determined using a plate reader (VersaMax ELISA Microplate Reader, Molecular Devices, Sunnyvale, CA, USA). At least three replicate wells were assayed for each condition, and the S.D. was determined.

### Gene expression profiling

Whole genome expression profiling of CMT93 cells under stimulation with rPostn (2 μg/ml) for 72 hours was performed with four biological replicates. RNA from CMT93 cells was purified using RNeasy Mini Kits and QIAshredder columns (Qiagen, Canada). Microarray analysis was performed using SurePrint G3 mouse Gene Expression 8 × 60 K Microarray Kits (Agilent Technologsies, CA) and the Low Input Quick Amp Labeling Kit (Agilent Technologies) according to the manufacturer's protocols. After hybridization and washing, the processed slides were scanned with an Agilent Microarray Scanner (Agilent Technologies), and the acquired array images were analyzed using the Agilent Feature Extraction Software (Agilent Technologies). Normalization and subsequent data processing were performed using R statistical software. The obtained gene expression data were expressed in a logarithmic scale, and heat maps were generated using the weighted average difference (WAD) algorithm [14].

### Histology and scoring

Histologic scoring was performed by an investigator blinded to the project based on previously described criteria [15]. 0 = normal; 1 = moderate mucosal inflammation without erosion and ulcer; 2 = severe mucosal inflammation with erosion; 3 = severe mucosal inflammation with ulcer (< 1 mm); 4 = severe mucosal inflammation with ulcer (1–3 mm); 5 = severe mucosal inflammation with ulcer (> 3 mm).

### Statistical analysis

Statistical analysis was performed using GraphPad Prism vision 6.0 g (GraphPad Software, La Jolla, CA, USA). The differences between two groups were analyzed by the unpaired *t*-test. The *P*-values < 0.05 were considered to be statistically significant.

## SUPPLEMENTARY MATERIALS FIGURES



## Abbreviations

7-AAD: 7-Amino-Actinomycin D
AOM: azoxymethane
CAF: cancer-associated fibroblast
CCD: charge coupled device
CRC: colorectal cancer
cDNA: complementary deoxyribonucleic acid
DMEM: Dulbecco's modified Eagle's medium
DSS: dextran sulfate sodium
ECL: enhanced chemiluminescence
EGF: epidermal growth factor
ELISA: enzyme linked immunosorbent assay
ERK: extracellular signal-regulated kinase
FACS: fluorescence activated cell sorting
FBS: fetal bovine serum
FITC: fluorescein isothiocyanate
GAPDH: glyceraldehyde 3-phosphate dehydrogenase
IHC: immunohistochemistry
MAPK: mitogen-activated protein kinase
MTT: 3-(4,5-dimethylthiazole-2-yl)-2
PAGE: poly-acrylamide gel electrophoresis
PBS: phosphate-buffered saline
Postn: periostin
PVDF: polyvinylidene difluoride
RNA: ribonucleic acid
RPMI: Roswell Park Memorial Institute medium
SDS: sodium dodecyl sulfate
TGF-ß: transforming growth factor-β
TNF: tumor necrosis factor
TUNEL: TdT-mediated dUTP nick end labeling
UC: ulcerative colitis
VEGF: vascular endothelial growth factor
