# Supplementary material for: Periostin attenuates tumor growth by inducing apoptosis in colitis-related colorectal cancer
Source: Oncotarget. 2018 Apr 13;9(28):20008–17. doi: 10.18632/oncotarget.25026 (PMC5929442; doi:10.18632/oncotarget.25026)
Supplement: Supplementary file 1 [file oncotarget-09-20008-s001.pdf]

Periostin attenuates tumor growth by inducing apoptosis in colitis-related colorectal cancer

SUPPLEMENTARY MATERIALS

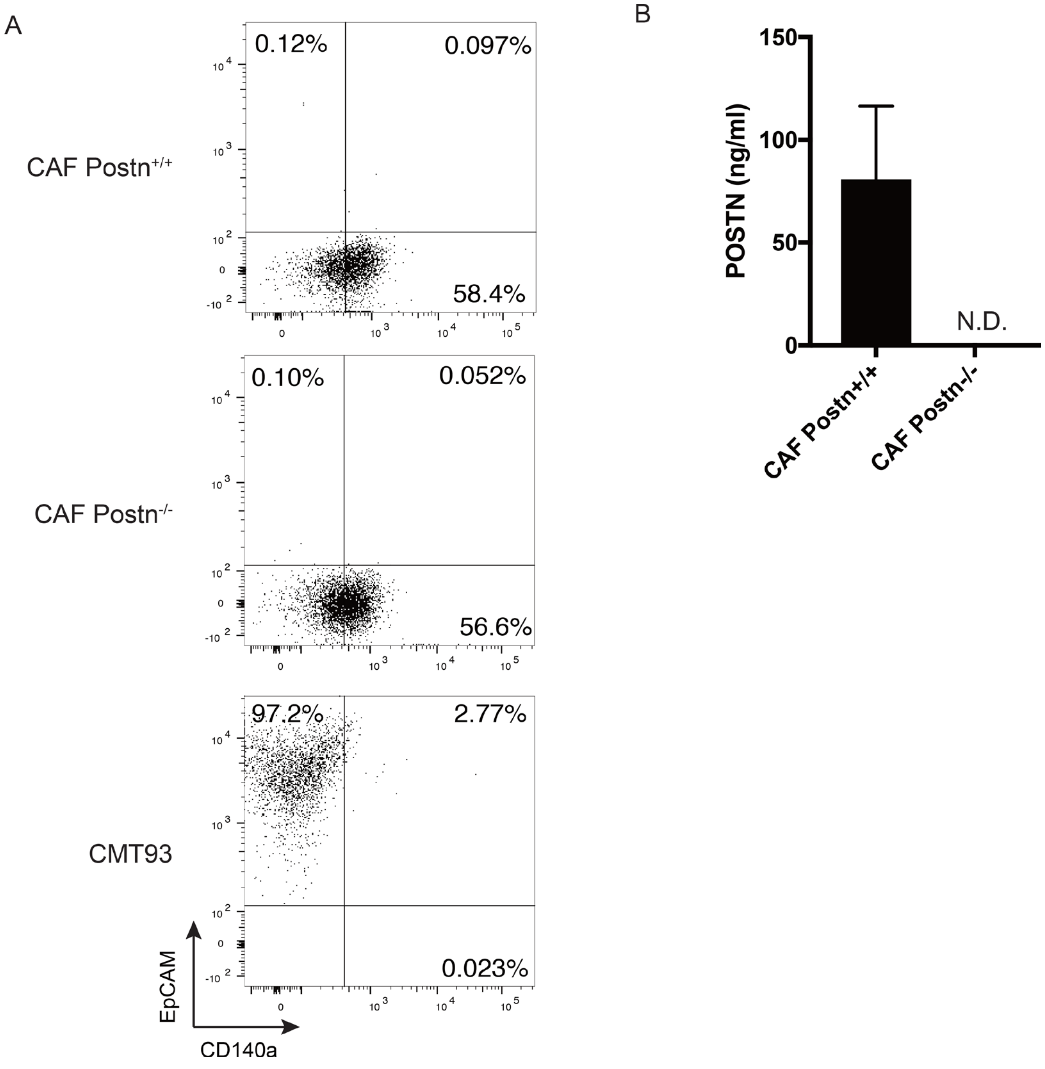

Supplementary Figure 1: Establishment of cancer-associated fibroblasts. (**A**) Flow cytometry analysis of established cancer-associated fibroblasts using FITC anti-EpCAM (BIolegend, clone G8.8) and APC anti-CD140a (Biolegend, clone APA5). CMT93 cells were also stained as a representative epithelial cells. (**B**) POSTN concentration in the supernatant of CAF was determined by ELISA assay according to the manufacture's instruction (R&D systems). N.D., not detected.

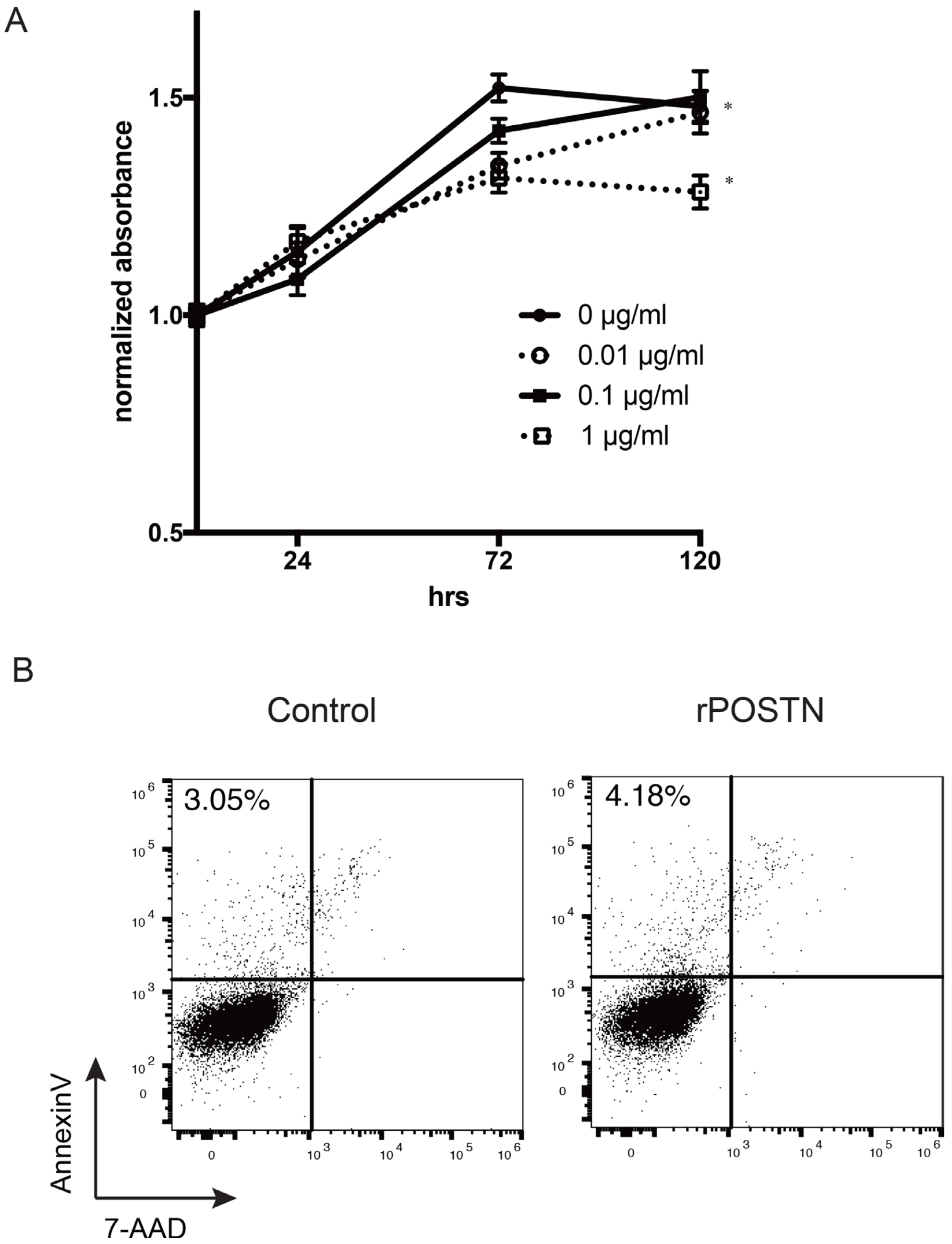

Supplementary Figure 2: Postn induces apoptosis and inhibits cell growth in colon-26 cell line. (**A**) Proliferation of colon-26 cells under the stimulation of rPostn assessed using MTT assay. $^{*}P < 0.01$ (0 vs 1 µg/ml). (**B**) Representative dot plots of AnnexinV and 7-AAD staining in Postn-treated or control colon-26 cells are shown.

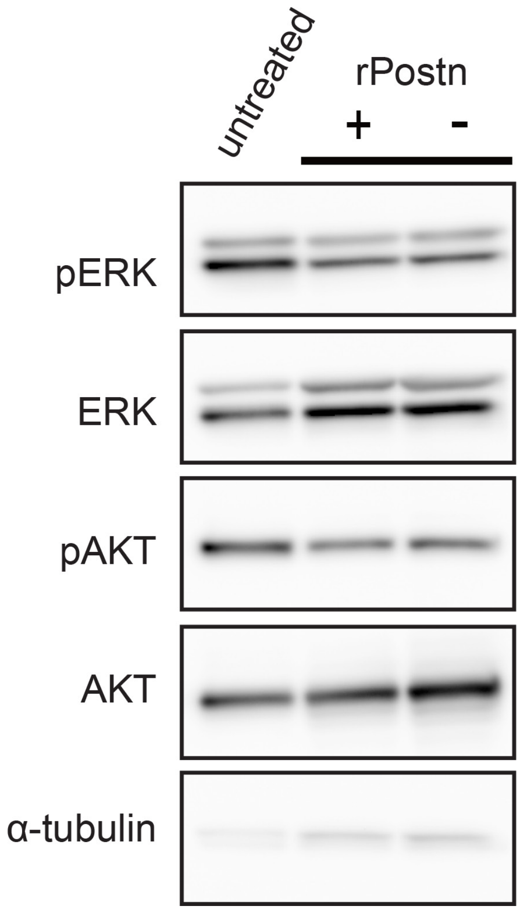

Supplementary Figure 3: Intracellular growth signaling was not altered in the presence of Postn. Western blot analysis of ERK, AKT, and their phosphorylation forms (pERK1/2 (Thr202/Tyr204), pAKT (Ser473)) was performed with CMT93 cells incubated for 24 hrs in 0.1% FBS-containing medium and then treated with rPostn.
